# Functional outcome in patients with traumatic or hemorrhagic brain injuries undergoing decompressive craniectomy versus craniotomy and 6-month rehabilitation

**DOI:** 10.1038/s41598-023-37747-0

**Published:** 2023-06-30

**Authors:** Valeria Pingue, Diego Franciotta

**Affiliations:** 1 Neurorehabilitation and Spinal Unit, Istituti Clinici Scientifici Maugeri IRCCS, Via Maugeri 4, 27100 Pavia, Italy; 2 IRCCS Mondino Foundation, Pavia, Italy

**Keywords:** Diseases, Neurology, Signs and symptoms

## Abstract

Decompressive craniectomy (DC) and craniotomy (CT) to treat increased intracranial pressure after brain injury are common but controversial choices in clinical practice. Studying a large cohort of patients with traumatic brain injury (TBI) and hemorrhagic stroke (HS) on rehabilitation pathways, we aimed to determine the impact of DC and CT on functional outcome/mortality, and on seizures occurrence. This observational retrospective study included patients with either TBI, or HS, who underwent DC or CT, consecutively admitted to our unit for 6-month neurorehabilitation programs between January 1, 2009 and December 31, 2018. Neurological status using Glasgow Coma Scale (GCS), and rehabilitation outcome with Functional Independence Measure, both assessed at baseline and on discharge, post-DC cranioplasty, prophylactic antiepileptic drug use, occurrence of early/late seizures, infectious complications, and death during hospitalization were evaluated and analyzed with linear and logistic regression models. Among 278 patients, DC was performed in 98 (66.2%) with HS, and in 98 (75.4%) with TBI, whilst CT in 50 (33.8%) with HS, and in 32 (24.6%) with TBI. On admission, GCS scores were lower in patients treated with CT than in those with DC (HS, p = 0.016; TBI, p = 0.024). Severity of brain injury and older age were the main factors affecting functional outcome, without between-group differences, but DC associated with worse functional outcome, independently from severity or type of brain injury. Unprovoked seizures occurred post-DC cranioplasty more frequently after HS (OR = 5.142, 95% CI 1.026–25.784, p = 0.047). DC and CT shared similar risk of mortality, which associated with sepsis (OR = 16.846, 95% CI 5.663–50.109, p < 0.0001), or acute symptomatic seizures (OR = 4.282, 95% CI 1.276–14.370, p = 0.019), independently from the neurosurgery procedures. Among CT and DC, the latter neurosurgical procedure is at major risk of worse functional outcome in patients with mild-to-severe TBI, or HS undergoing an intensive rehabilitation program. Complications with sepsis or acute symptomatic seizures increase the risk of death.

## Introduction

Decompressive craniectomy (DC) and craniotomy (CT) are common surgical approaches used to resolve raised intracranial pressure due to mass effect after acute brain injury (ABI)^1^. In DC, a large section of the cranial vault is removed, and cranial reconstruction is performed a few weeks to months later (cranioplasty)^2^. Instead, in CT the bone flap is fixed back on the skull after the evacuation of hematoma^3^.

Previous studies examined mortality and neurologic outcome after DC and CT in patients with ABI with variable results, although there is more evidence suggesting that DC is associated with worse outcome in comparison with CT^4–6^. However, which of the two yields better outcome, and how to manage residual morbidity remain open questions^4^.

Seizures are common complications of these surgical procedures^7^. In particular, brain surgery after ABI can result in the occurrence of both early and late seizures^8^. Acute symptomatic seizures (ASS) are associated with acute, and possibly reversible, neuronal dysfunction. In contrast, late-onset seizures are considered unprovoked seizures (US) that arise from structural changes connected with an enduring predisposition to generate seizures, being de facto as an epilepsy condition after brain injury^9^. Both seizures and epilepsy can potentially affect neurological outcome in traumatic brain injury (TBI)^10^, ischemic or hemorrhagic stroke^11^, and acute neurosurgery^12^. Therefore, the prescription of prophylactic antiepileptic drugs (AEDs) is common in subjects with ABI. This empirical practice is in contrast to the current guidelines recommending the use of AEDs during the first week after TBI only^13^, whilst there is no evidence to support seizures prophylaxis after this time frame window, nor after ischemic or hemorrhagic strokes^11, 14^.

In this retrospective cohort study on a large number of rehabilitation patients followed from the post-acute phase up to 6 months after TBI or hemorrhagic stroke (HS), we aimed to compare DC and CT to assess differences in terms of induction of post-surgery seizures and of long-term functional outcome.

## Materials and methods

### Patients

For this observational retrospective study, we included patients with TBI or HS, consecutively admitted to the Neurorehabilitation Unit of the ‘Istituti Clinici Scientifici Maugeri’ of Pavia, Italy, between January 1, 2009 and December 31, 2018.

The inclusion criteria were: (1) age ≥ 18 years; (2) diagnosis of HS or TBI; (3) admission to our rehabilitation unit within one month from ABI to continue clinical care and rehabilitation programs started at the intensive care unit; (4) DC or CT performed at the acute care settings as a part of their neurocritical management; (5) at least 6 months of observation before discharge.

Patients were excluded if: (1) they suffered from pre-existing brain injuries, or any other neurological disease; (2) they had a history of epilepsy and/or concurrent use of AEDs; (3) they underwent DC or CT for any reason other than TBI or HS; (4) detailed data on acute care were unavailable.

The study design was in conformity with the ethical guidelines of the Declaration of Helsinki and was approved by the local Ethical Committee (ICS Maugeri, ref. 2214 CE). All participants or their legal guardians signed a written informed consent.

### Variables, data sources and measurements

The following data were collected from electronic clinical records: age at occurrence of injury, sex, type of neurosurgical procedures (DC/CT), cranioplasty after DC, neurological and functional assessments, occurrence of sepsis, acute hypoxemic respiratory failure (AHRF) requiring oxygen or mechanical ventilation, occurrence of seizures, use of AEDs, death during hospitalization. As for seizures, any paroxysmal event that occurred during hospitalization, either described by patients, or eye witnessed, was examined by clinicians. Epileptic seizures were diagnosed on the basis of clinical features and EEG findings. In accordance with the International League Against Epilepsy (ILAE) criteria, seizures were classified as ASS if they occurred within 1–7 days after ABI or related neurosurgery, or US if they occurred more than 7 days^9^. Seizures prophylaxis with AEDs was prescribed in either acute setting care, or rehabilitation settings. The Glasgow Coma Scale (GCS), and the Functional Independence Measure (FIM) scale were used to evaluate neurological and functional outcomes, respectively. GCS assesses the degree of neurological impairment severity (13-to-15 scores indicate “mild”, 9-to-12 “moderate”, 8-or-less “severe” brain injury)^15, 16^. The FIM scale, focused on patients’ independence in activities of daily living, evaluates the level of disability with 13 motor and 5 cognitive items. The total score ranges from 18 (complete dependence/total assistance) to 126 (complete independence)^17^. GCS and FIM scales were administered on admission to (T0), and on discharge from (T1) the rehabilitation unit. All participants underwent an inpatient intensive neurorehabilitation program consisting of individual 3-h daily treatment cycles, 6 days per week. During the study period, there was no therapeutic limitation by relatives due to the patient's presumed will.

### Statistical analysis

Categorical variables were expressed as absolute number and percentage, and compared with Chi-square test. Continuous variables were expressed as median and interquartile ranges (IQR). Multivariate linear regression analysis was used to evaluate the predictive role of DC, CT, cranioplasty, sepsis, AHRF, seizures, and AED therapy on rehabilitation outcome. The multilinear models included FIM scores on discharge (T1), as dependent variables, and age, sex (male = 0, female = 1), GCS score (classified as mild = 1, moderate = 2, and severe = 3) on admission (T0), presence/absence of seizures, and of sepsis or AHRF (No = 0, Yes = 1), neurosurgical procedure (CT = 0, DC = 1), and etiology of injury (TBI = 0, HS = 1) as independent variables. Coefficients of determination (R^2^), β coefficients, and p-values obtained from the models were reported. A number of different models were tested to avoid collinearity. The models achieving the highest R^2^ were reported. Kaplan–Meier analysis was used to estimate survival rates between patients who underwent DC/CT, log-rank test to examine the difference in survival probability of the two neurosurgical procedures. Among the considered variables (sex, age, GCS at T0, seizures, sepsis, acute respiratory failure, ARDS, prophylaxis with AEDs, neurosurgical procedure, and etiology of injury), multivariate logistic regression analysis was used to identify the potential risk factors of mortality, ASS, and US. Odds ratio (OR), 95% confidence interval (95% CI), and related significant values obtained from the regression analysis were reported. Statistical significance was set at 5%. Statistical analyses were performed using SPSS Statistics 21 (IBM Corporation, Somers, NY, USA).

## Results

### Demographic and clinical characteristics

**Figure 1:**
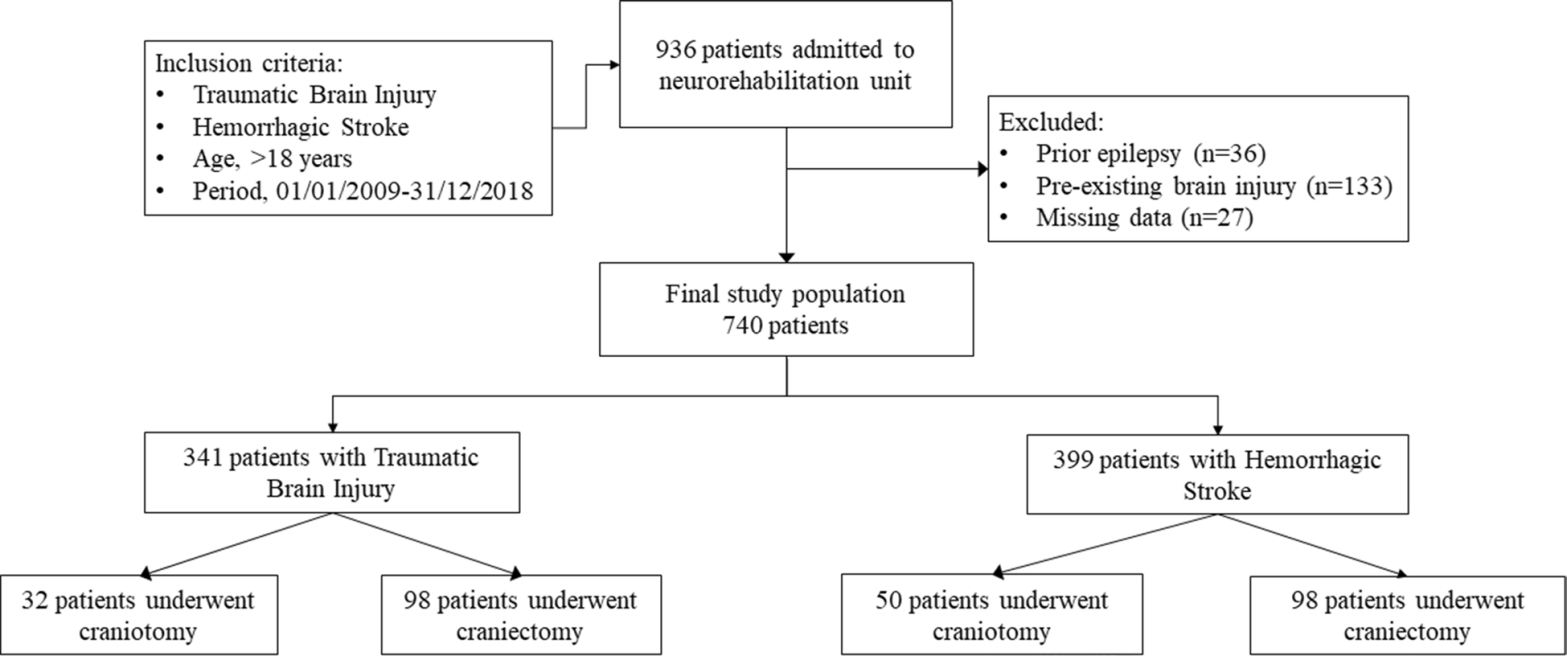
Flow diagram of patients with acute brain injury enrolled in the study.

We enrolled 740 eligible patients with mild-to-severe ABI, namely, mild 256 (34.6%); moderate 306 (41.4%); and severe 178 (24.0%), as a result of TBI (341; 46.1%), or HS (399; 53.9%). We enrolled 740 eligible patients with mild-to-severe ABI, namely, mild 256 (34.6%); moderate 306 (41.4%); and severe 178 (24.0%), as a result of TBI (341; 46.1%), or HS (399; 53.9%). Among these patients, 278 underwent DC or CT after both types of injury, the former more frequent than the latter (p < 0.0001). In particular, DC was performed in 98 (66.2%) with HS, and in 98 (75.4%) with TBI, whilst CT in 50 (33.8%) with HS, and in 32 (24.6%) with TBI (Fig. 1). Overall, 125 patients (63.8%) underwent cranioplasty after DC during the 6-month rehabilitation period.

The patients that had undergone CT presented significantly less severe GCS scores at baseline than those who required DC, irrespective from etiology of injury (HS, p = 0.016; TBI, p = 0.024). Prophylactic AEDs were prescribed more frequently after HS than after TBI (p = 0.036). The patients that developed seizures were 37 (25%) after HS, and 37 (28.5%) after TBI. During hospitalization, severe sepsis occurred in 33 patients (11.9%), AHRF in 56 patients (20.1%). No difference in frequency of seizures occurrence, nor of clinical complications was detected between the two neurosurgery procedures after HS, or TBI within the 6-month rehabilitation period. Table 1 shows the clinical and demographic characteristics of the patients that underwent the neurosurgery procedures, subdivided according to etiology of the brain injury.

**Table 1:** Clinical and demographic characteristics of patients with traumatic brain injury, or hemorrhagic stroke who underwent neurosurgery procedures.

	Neurosurgery patients n = 278							
	Hemorrhagic stroke			*P value*	Traumatic brain injury			*P value*
	Whole population n = 148 (53.2%)	CT n = 50 (33.8%)	DC n = 98 (66.2%)		Whole population n = 130 (46.8%)	CT n = 32 (24.6%)	DC n = 98 (75.4%)	
	n (%)	n (%)	n (%)		n (%)	n (%)	n (%)	
Age (years)								
≤ 65	72 (48.6)	19 (38.0)	53 (54.1)	0.082	70 (53.8)	13 (40.6)	57 (58.2)	0.103
> 65	76 (51.4)	31 (62.0)	45 (45.9)		60 (46.2)	19 (59.4)	41 (41.8)	
Sex								
Male	78 (52.7)	29 (58.0)	49 (50.0)	0.388	95 (73.1)	24 (66.7)	71 (72.4)	0.526
Female	70 (47.3)	21 (42.0)	49 (50.0)		35 (26.9)	8 (33.3)	27 (27.6)	
GCS score on admission								
Mild	30 (20.2)	16 (32.0)	14 (14.3)	**0.016**	36 (27.7)	14 (43.7)	22 (22.5)	**0.024**
Moderate	63 (42.6)	24 (48.0)	39 (39.8)	0.381	48 (36.9)	10 (31.3)	38 (38.8)	0.529
Severe	55 (37.2)	10 (20.0)	45 (45.9)	**0.002**	47 (36.2)	8 (25.0)	39 (39.8)	0.144
AHRF	30 (20.3)	9 (18.0)	21 (21.4)	0.671	26 (20.0)	6 (18.8)	20 (20.4)	0.999
Sepsis	20 (13.5)	5 (10.0)	15 (15.3)	0.167	13 (10.0)	2 (6.3)	11 (11.2)	0.518
Patients with seizures	37 (25.0)	9 (18.0)	28 (28.6)	0.228	37 (28.5)	7 (21.9)	30 (30.6)	0.377
ASS	12 (8.1)	1 (2.0)	11 (11.2)	0.060	12 (9.2)	4 (12.5)	8 (8.2)	0.488
US	20 (13.5)	7 (14.0)	13 (13.3)	0.999	19 (14.6)	2 (6.3)	17 (17.3)	0.156
ASS + US	5 (3.4)	1 (2.0)	4 (4.1)	0.662	6 (4.6)	1 (2.0)	5 (5.1)	0.664
Prophylaxis with AEDs	62 (41.9)	27 (54.0)	35 (35.7)	**0.036**	51 (39.2)	16 (50.0)	35 (35.7)	0.210
Mortality within 6 months	16 (10.8)	4 (8.0)	12 (12.2)	0.579	27 (20.8)	4 (12.5)	23 (23.5)	0.218

Comparison between populations, expressed as absolute number and percentage, was performed with χ^2^ test. Significant associations in bold character.

*AED* antiepileptic drug, *AHRF* acute hypoxemic respiratory failure, *ASS* acute symptomatic seizures, *CT* craniotomy, *DC* decompressive craniectomy, *GCS* Glasgow coma scale, *US* unprovoked seizures.

### Functional outcome, mortality and seizures

Table 2 shows the results obtained with multilinear regression models for independent predictors of functional outcome in the neurosurgery patients.

**Table 2:** Predictors of functional outcome measured with FIM on discharge (T1) in patients with traumatic brain injury, or hemorrhagic stroke who underwent neurosurgery procedures.

Independent variables	FIM T1 (R^2^ = 0.559)	
	Beta	*P values*
Sex (M = 0, F = 1)	− 0.34	0.480
Age > 65 years	− 0.19	** < 0.0001**
Glasgow Coma Scale score on admission (mild = 1, moderate = 2, severe = 3)	− 0.64	** < 0.0001**
Acute symptomatic seizures	− 0.06	0.194
Unprovoked seizures	− 0.06	0.149
Acute hypoxemic respiratory failure	− 0.03	0.525
Sepsis	− 0.14	**0.003**
Neurosurgery procedure (DC = 1, CT = 0)	− 0.13	**0.031**
Cranioplasty	0.05	0.369
Etiology of injury (HS = 1, TBI = 0)	− 0.05	0.281

Significant associations in bold character.

*CT* craniotomy, *DC* decompressive craniectomy, *FIM* functional independence measure, *HS* hemorrhagic stroke, *TBI* traumatic brain injury.

**Figure 2:**
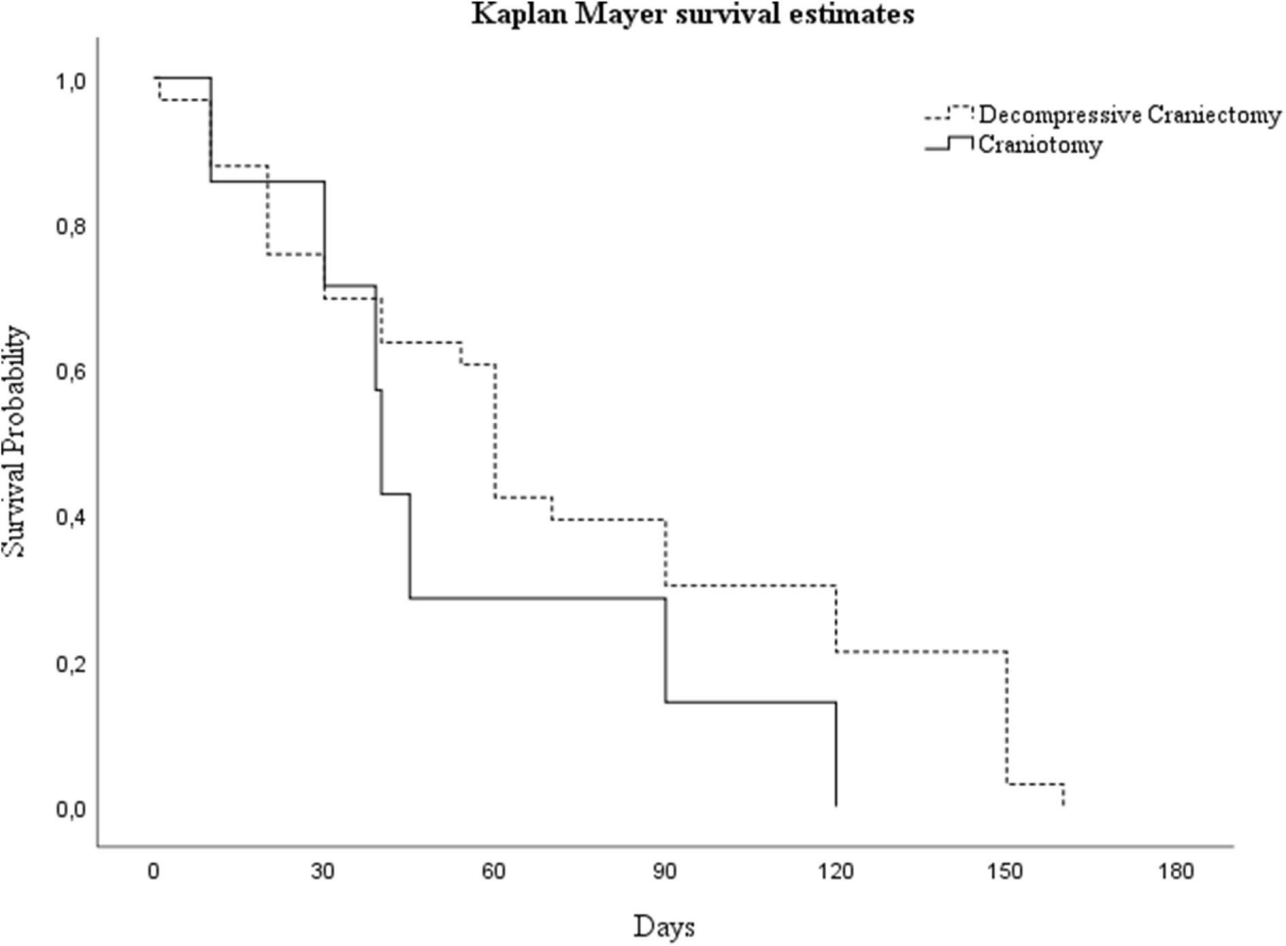
Kaplan–Meier curves of survival probability in the two neurosurgical groups.

Age over 65 years (p < 0.0001), and worse GCS scores on admission (p < 0.0001), occurrence of sepsis (p = 0.003), and DC (p = 0.031) predicted poor functional outcomes on discharge, independently from the considered variables. Figure 2 shows curves of survival probability in the two neurosurgical procedure groups (Kaplan–Meier analysis).

Survival rate at 6 months was higher in post-DC (2.839, 95% CI 54.435–65.565), than in post-CT (1.309, 95% CI 37.434–42.566) patients, but the difference was not statistically significant (p = 0.187).

Multivariate logistic regression analysis was conducted to evaluate the potential role of seizures, AHRF, sepsis, neurosurgical procedures, severity and type of brain damage in influencing mortality in neurosurgical patients during the 6-month period of rehabilitation (Table 3).

**Table 3:** Potential risk factors for mortality during inpatient rehabilitation in patients with traumatic brain injury, or hemorrhagic stroke who underwent neurosurgery procedures.

	Death during rehabilitation	
	OR (95% CI)	*P value*
Sex (M = 0, F = 1)	0.036 (0.343–0.126)	**0.036**
Age > 65 years	4.907 (2.177–11.062)	** < 0.0001**
Glasgow coma scale score on admission (mild = 1, moderate = 2, severe = 3)	3.080 (1.588–5.973)	**0.001**
Acute symptomatic seizures	4.282 (1.276–14.370)	**0.019**
Unprovoked seizures	0.412 (0.094–1.809)	0.240
Acute hypoxemic respiratory failure	0.640 (0.248–1.647)	0.354
Sepsis	16.846 (5.663–50.109)	** < 0.0001**
Neurosurgery procedure (DC = 1, CT = 0)	1.613 (0.548–4.746)	0.386
Cranioplasty	0.723 (0.273–1.914)	0.514
Etiology of injury (HS = 1, TBI = 0)	0.359 (0.142–0.906)	**0.030**

Significant associations in bold character.

*CT* craniotomy, *DC* decompressive craniectomy, *HS* hemorrhagic stroke, *TBI* traumatic brain injury.

Older age at diagnosis (p < 0.0001), severity of brain injury (p = 0.001), ASS (p = 0.019) and sepsis (p < 0.0001) emerged as the major predictors of mortality in the neurosurgical patients. Risk of death was inversely associated with female sex and HS. The regression analysis did not detect differences between DC and CT in mortality risk.

Finally, a multivariate logistic regression analysis was conducted to identify the potential risk factors for ASS and US development within the 6-month inpatient rehabilitation period (Table 4).

**Table 4:** Potential risk factors for seizures occurrence in patients with traumatic brain injury, or hemorrhagic stroke who underwent neurosurgery procedures.

Covariates	Acute symptomatic seizures			Unprovoked seizures		
	OR	95% CI	*P value*	OR	95% CI	*P value*
Hemorrhagic stroke						
Sex (M = 0, F = 1)	3.097	0.890–10.781	0.059	1.416	0.517–3.880	0.499
Age > 65 years	1.556	0.457–5.300	0.480	1.153	0.435–3.058	0.775
Glasgow coma scale on admission	1.678	0.688–4.094	0.255	0.580	0.279–1.204	0.144
Acute symptomatic seizures	n.a	n.a	n.a	2.009	0.548–7.367	0.200
Primary prophylaxis with AEDs	0.000	0.000-/	0.997	0.481	0.165–1.404	0.160
Neurosurgery procedure (DC = 1, CT = 0)	2.055	0.383–11.032	0.401	0.456	0.057–1.363	0.118
Cranioplasty	5.735	1.126–23.564	0.118	5.142	1.026–25.784	**0.047**
Traumatic brain injury						
Sex (M = 0, F = 1)	1.055	0.344–3.241	0.925	0.760	0.260–2.220	0.616
Age > 65 years	0.727	0.248–2.130	0.561	0.473	0.178–1.260	0.134
Glasgow coma scale on admission	1.023	0.518–2.020	0.949	1.402	0.755–2.602	0.284
Acute symptomatic seizures	n.a	n.a	n.a	2.042	0.612–6.812	0.245
Primary prophylaxis with AEDs	0.000	0.000-/	0.997	0.512	0.168–1.561	0.239
Neurosurgery procedure (DC = 1, CT = 0)	0.789	0.234–2.661	0.702	2.584	0.685–9.742	0.161
Cranioplasty	1.308	0.337–5.081	0.187	1.356	0.482–3.820	0.564

Significant associations in bold character.

*AED* antiepileptic drug, *CT* craniotomy, *DC* decompressive craniectomy, *n.a.* not applicable.

Cranioplasty was a risk factor for US in HS group (p = 0.047). Of note, in this contest, the prescription of prophylactic therapy was not effective in preventing occurrence of seizures. No further significant association was observed between risk for ASS or US development and the covariates considered in the analysis. In particular, there was no difference between DC or CT procedures and the risk of seizures.

## Discussion

The choice of either DC, or CT to reduce intracranial pressure after TBI, or HS is at neurosurgeon’s discretion, in the absence of specific guidelines^18^. These procedures can worsen the functional outcome, and increase the chance of seizures occurrence, which is very high over the 6-month rehabilitation period following the brain insult, when patients can potentially achieve the maximum functional recovery^19^. The main finding of our study indicates that DC conveys worse functional outcome after six months of intensive rehabilitation program, independently from both the etiologies, and even after adjusting for confounding variables, such as severity of the brain injury, age, and occurrence of neurological and clinical comorbidity (seizures, sepsis, ARHF). This surgical repair of the skull has been associated with a high rate of post-operative complications and poor outcome^20^. On the other hand, DC and CT shared similar risks of mortality, notwithstanding that the proportion of patients with severe GCS scores at baseline in DC group was higher than in CT group. The results of a recent clinical trial suggest instead that DC and CT share the same outcome, evaluated at 6 and 12 months after acute subdural hematoma^21^. However, the case series and the aim of the two studies were quite different, as our cohort was composed of post-ABI selected patients discharged from neurosurgery wards, with outcomes that could be treated with rehabilitation. Post-rehabilitation functional outcome was assessed by the same medical team on admission and on discharge in our study, whereas by postal questionnaires, or telephone interviews in Hutchinson et al.’s study^21^. As expected, older age and worse neurologic presentations on admission were other main predictors of poor functional outcome and mortality in our study. Sepsis was another risk factor for death. The rates of death due to life threatening complication were relatively high in our patients, as expected in neurological intensive care units and in rehabilitation settings^22, 23^, and especially in patients with severe comorbidities and impaired functional status^24^.

Previous studies examining mortality and outcome of DC vs CT reported contrasting results. Some suggest that DC has higher mortality rates and worse functional outcome when compared to CT^4–6^, while others find no significant outcome difference between the two neurosurgery procedures^3, 18^. The main reasons for these discrepancies could be due to the heterogeneity of populations and settings, as well as the lack of adjustment for important covariates that limits the interpretation of the results.

As for the topic of seizures, neither DC, nor CT emerged as risk factors of seizures development, whereas the occurrence of ASS increased four-fold the risk of mortality during the rehabilitation period. Of note, the prescription of prophylactic therapy was not effective in preventing occurrence of early or late seizures in both TBI/HS groups. ASS has a typical temporal association with brain injury, likely resulting from an acute hyperexcitability of the damaged neural tissue that contributes to lower the epileptogenic threshold. In the acute phase, patients with ASS are subjected to higher mortality rates, although the long-term rates are similar to those following US^25^. ASS might also worsen the neurological damage in the perioperative course of ABI-related neurosurgery due to the sudden change in blood flow and intracranial pressure^26^. As in our cohort, post-operative seizures have been documented in a high proportion of ABI patients who underwent neurosurgery^27^, with detrimental effects on mortality and outcome^12^. This has led to the evidence-based recommended early starting of antiepileptic medications in post-TBI patients to prevent ASS^13, 28^. By inference, short-term prophylaxis with AEDs could be appropriate in patients scheduled for neurosurgery after HS, or TBI. However, our data did not allow us to draw conclusions on the efficacy of AEDs in early seizures prevention in these patients. To date, the few prospective and randomized controlled trials that addressed this topic yielded no consensus to guide medical decision-making approach to patients requiring neurosurgery^29^.

### Limitations

This study has some limitations. First, the retrospective design implies review of charts not originally aimed at collecting data for research, with selection and recall biases, and possibility of missing information. For instance, descriptions of the intracranial lesions due to injuries or bleedings were unavailable. However, the sample size is very large, and the well-characterized cohort of patients was hospitalized in a third-level referral center. Second, the choice of DC or CT was at neurosurgeon’s discretion, likely taking into account severity of clinical and radiological pictures, and there was no randomization (selection bias). Third, in contrast to previous studies^3, 18^, in our cohort DC was performed more frequently than CT. This likely could depend on the high percentage of patients with moderate-severe ABI usually admitted to our unit. To overcome these confounding variables, we built a multivariable logistic regression model, analyzing different covariates to better define the role of DC, or CT in influencing functional recovery.

## Conclusions

Patients requiring primary DC had a higher risk for poor neurological outcome and morbidity in comparison with patients undergoing CT after intensive rehabilitation program. DC and CT share similar risks of mortality rates after haemorrhagic, or traumatic brain injury. Sepsis and ASS occurrence seem to increase the risk of mortality, independently from the neurosurgery procedure.

## Data Availability

The data associated with the paper are not publicly available but are available from the corresponding author on reasonable request.
